# A Dual Blind Watermarking Method for 3D Models Based on Normal Features

**DOI:** 10.3390/e25101369

**Published:** 2023-09-22

**Authors:** Qijian Tang, Yanfei Li, Qilei Wang, Wenqi He, Xiang Peng

**Affiliations:** 1 Key Laboratory of Optoelectronic Devices and System of Ministry of Education and Guangdong Province, College of Physics and Optoelectronic Engineering, Shenzhen University, Shenzhen 518060, China; 2 Technology Department, Heyuan Shenzhen University Bay Institute, Heyuan 517099, China

**Keywords:** dual digital watermarking, three-dimensional model, blind detection, normal feature

## Abstract

Digital watermarking technology is an important means to effectively protect three-dimensional (3D) model data. Among them, “blind detection” and “robustness” are key and difficult points in the current research of digital watermarking technology based on 3D models. In order to realize the blind detection of a watermark and improve its robustness against various common attacks at the same time, this paper proposes a dual blind watermarking method based on the normal feature of the centroid of first-ring neighboring points. The local spherical coordinate system is constructed by calculating two different normal vectors, and the first pattern watermark and the second random binary sequence watermark are embedded, respectively. The experimental results show that this method can not only realize the blind detection of dual watermarks, but also have the ability to resist common attacks such as translation, rotation, scaling, cropping, simplification, smoothing, noise, and vertex reordering to a certain extent.

## 1. Introduction

Digital watermarking technology has emerged as an effective tool for safeguarding copyrights, making it a significant topic in the realm of digital multimedia research [1,2]. Currently, the majority of watermarking research is centered around images, audio, and videos [3,4,5,6], with relatively fewer studies addressing watermarking in the context of 3D digital models. It is widely recognized that in recent years, 3D models have found extensive applications in diverse fields such as industrial manufacturing, urban planning, architectural design, healthcare, cultural heritage preservation, film, gaming, and virtual reality. Hence, research focused on watermarking techniques tailored to 3D models holds vital scientific and commercial value. However, when compared to traditional multimedia watermarking techniques for text, images, audio, and videos, the exploration of watermarking techniques for 3D models is still in its developmental stage. This is primarily due to four factors: the non-linear nature of 3D model data, the non-uniqueness of representation methods, a lack of natural parameterization decomposition techniques, and the increased diversity and complexity of potential attack methods [7].

Watermark algorithms can be categorized into blind watermarking and non-blind watermarking based on whether the original 3D model is needed for watermark detection, with the latter requiring the original model while the former does not [8]. Evidently, blind watermarking technology significantly streamlines the watermark detection process, thereby offering greater practical application value. Researchers worldwide have undertaken relevant research on 3D digital watermarking. Among them are some groundbreaking and classic 3D model digital watermarking algorithms, listed as follows. In 1997, Ohbuchi and colleagues from IBM Tokyo Research Laboratory published the first report on 3D digital model watermarking at the ACM Multimedia International Conference [9]. This work introduced various 3D model watermarking algorithms like the Triangle Similarity Quadruple (TSQ) and the Tetrahedral Volume Ratio (TVR) methods, although their robustness was limited. Kanai and Date, from Hokkaido University in Japan, introduced a multiresolution analysis-based 3D model watermarking method rooted in wavelet transformation and polygonal models [10]. Benedens et al. embedded digital watermarks by modifying the surface normal vectors of 3D models, demonstrating some resistance against simplification attacks [11]. Praun and colleagues proposed a spread-spectrum watermarking algorithm based on interpolation basis functions in the same year, which possessed a certain level of robustness, but required the original model for watermark detection [12]. Yu and his team embedded watermarks by altering the distance from the model vertices to the model center [13]. Subsequent researchers made enhancements based on their algorithms [14]. Harte and Bors introduced a blind watermarking algorithm for 3D mesh models. This algorithm established ellipsoids based on vertices and their first-ring neighbors, selecting vertices whose distances to neighbors were less than a specified threshold and altering their relative positions with the ellipsoids to embed the watermark [15,16]. Following that, Li and others proposed a 3D model watermarking algorithm based on spherical parameterization and harmonic analysis [17], while Cho and colleagues developed a watermarking algorithm adjusting vertex norm distributions based on the embedded watermark [18]. In recent years, the increasing demand for digital watermarking of 3D models has also led to the rapid development of this direction. Researchers have also published many excellent watermarking algorithms one after another. In 2017, Choi et al. proposed a solution for cropping attacks, aiming to address synchronization issues caused by cropping attacks by obtaining reference points from local model shapes [19]. This method evenly distributes watermark energy throughout the entire model, enhancing watermark invisibility. The following year, Jang et al. introduced a blind watermark algorithm based on consistency segmentation [20]. It relied on vertex norm consistency and employed stepwise analysis techniques to determine watermark schemes. However, this method requires embedding a sufficient number of vertices and is not suitable for small models. In 2019, Hamidi et al. proposed a three-dimensional model blind watermarking algorithm based on wavelet transform [21]. This algorithm embedded watermarks by modifying the vector norms of wavelet coefficients and exhibited good resistance against smoothness, additive noise, and similar transformation attacks. However, it requires further improvement to withstand severe cropping attacks and grid re-sampling, and it involves complex computations. To address cropping attacks, in 2020, Ferreira et al. published a watermarking algorithm for 3D point cloud models [22]. This algorithm embedded watermark information into the color data of point clouds through DFT transformation, and it demonstrated strong robustness against model cropping, noise, geometric, and other attacks. In comparison to blind watermarking, non-blind watermarking involves lower embedding difficulty and boasts stronger resistance against attacks [23]. However, non-blind watermarking algorithms not only require access to the original model for watermark embedding, but also entail complex preprocessing during watermark detection. Especially given the current immaturity of 3D model retrieval techniques and the continuous expansion of 3D model databases, the practical application of non-blind watermarking technology faces substantial limitations. Therefore, the pursuit of blind watermarking techniques tailored to 3D models holds significant practical significance [24,25,26,27]. Furthermore, the present emphasis on 3D model watermarking predominantly lies within the realm of single watermarking. Although these watermarks effectively secure carriers during regular usage, they often struggle to withstand an array of diverse attack methods. Consequently, the development of dual or even multiple watermarking techniques for 3D models is an urgently required research avenue [28,29]. This would elevate the robustness of watermarking algorithms in the face of various attack strategies. 

This paper presents a dual watermarking technique based on normal features. It begins by computing two distinct normal vectors for each vertex in a 3D model using its first-ring neighboring points and their centroid. Next, a local spherical coordinate system is established for the model vertices, utilizing the centroid point and the normal vectors. Subsequently, inspired by transformation domains, the discrete cosine domain coding values of the first watermark are integrated into the local spherical coordinates of the model vertices. Additionally, by considering statistical factors, the second watermark is embedded into the second-ring neighboring points through adjustments in their positions relative to the edges of the first-ring neighborhood. This dual watermarking method is designed to be mutually non-interfering and provides both invisibility and robustness, making it highly valuable for practical applications.

## 2. Algorithm Principle

**Figure 1:**
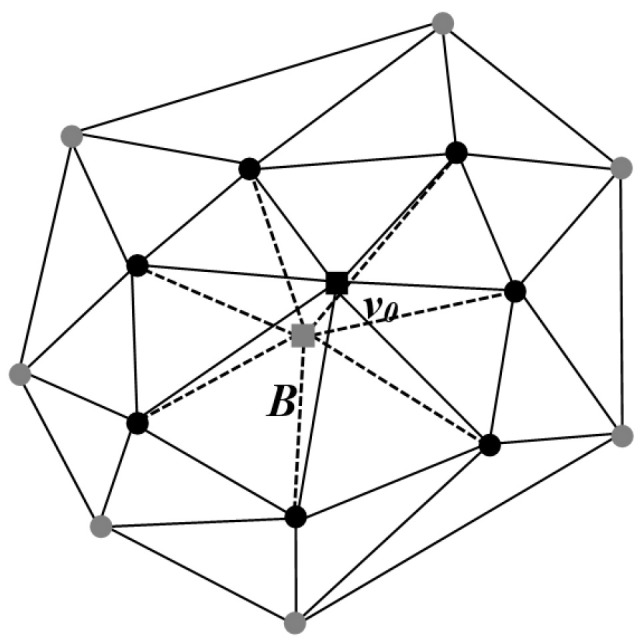
Local 3D model: ■ vertex of the model; ■ centroid point of the first-ring neighboring points; ● the first-ring neighboring points; ● the second-ring neighboring points.

In a 3D digital model, the mesh model *M* associated with each vertex can be represented as *M* = {*V_m_*, *K_n_*}. Here, *V_m_* denotes the set of vertices of *M*, where *m* represents the number of vertices in the mesh model; *K_n_* represents the collection of all topological connectivity relations of *M*; *n* represents the count of triangular faces in the mesh model; and the elements of *K_n_* fall into three types: vertices *v* = {*i*}, edges *e* = {*i*, *j*}, and facets *f* = {*i*, *j*, *k*}. If the edge {*i*, *j*} ∈ *K_n_*, the vertices {*i*} and {*j*} are mutually referred to as neighbors. The first-ring neighbors of vertex {*i*} are defined as *N*_1_(*i*) = {*j* | {*i*, *j*} ∈ *K_n_*}. The second-ring neighbors of vertex {*i*}, denoted as *N*_2_(*i*), refer to non-first-ring neighboring points that have a connection with the first-ring neighboring points *N*_1_(*i*). Moreover, the term “first-ring neighboring edge” is defined as the edge determined by the connecting relationship between two first-ring neighboring points (Figure 1).

The centroid B of the first-ring neighboring vertices of any mesh model vertex *v*_0_ can be determined by the following equation:

(1) $$B=\frac{1}{\left|N_{1}\left(v_{0}\right)\right|}\sum N_{1}\left(v_{0}\right)$$

where $\left|N_{1}\left(v_{0}\right)\right|$ represents the number of first-ring neighboring vertices. If the centroid *B* is connected to each of the first-ring neighboring vertices of vertex *v*_0_, it results in $\left|N_{1}\left(v_{0}\right)\right|$ triangles, which are collectively referred to as the set *T*(*B*). The following section will introduce two distinct centroid normal vectors based on these triangles.

**Figure 2:**
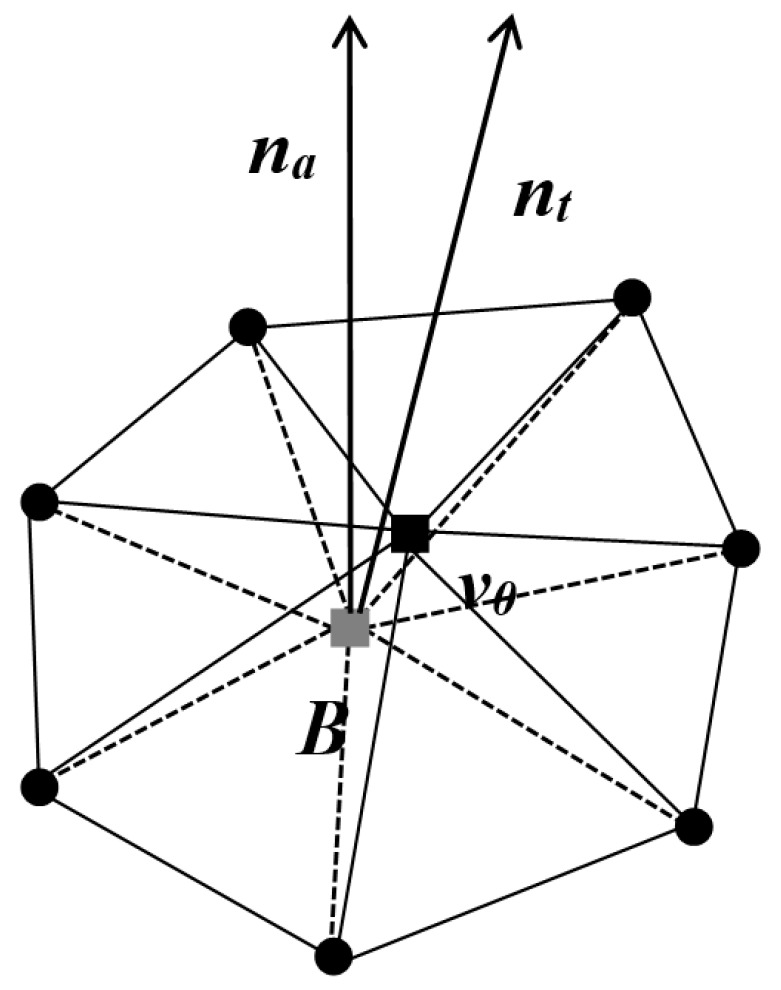
Normal $n_{t}$ and $n_{a}$: ■ vertex of the model; ■ centroid point of the first-ring neighboring points; ● the first-ring neighboring points.

As shown in Figure 2, the first type of centroid normal is defined by directly computing the average of the normal vectors of all triangles in *T*(*B*), denoted as $n_{t}$:

(2) $$n_{t}=\frac{1}{\left|N_{1}\left(v_{0}\right)\right|}\sum n_{i}$$

where $n_{i}$ represents the normal vector of the *i*-th triangle in the triangle set *T*(*B*).

The second type of centroid normal is determined collectively by the area and normal vector $n_{i}$ of each triangle in *T*(*B*), denoted as $n_{a}$:

(3) $$n_{a}=\frac{1}{\sum A_{i}^{2}}\sum n_{i}A_{i}^{2}$$

where *A_i_* denotes the area of the *i*-th triangle in the set of triangles *T*(*B*).

**Figure 3:**
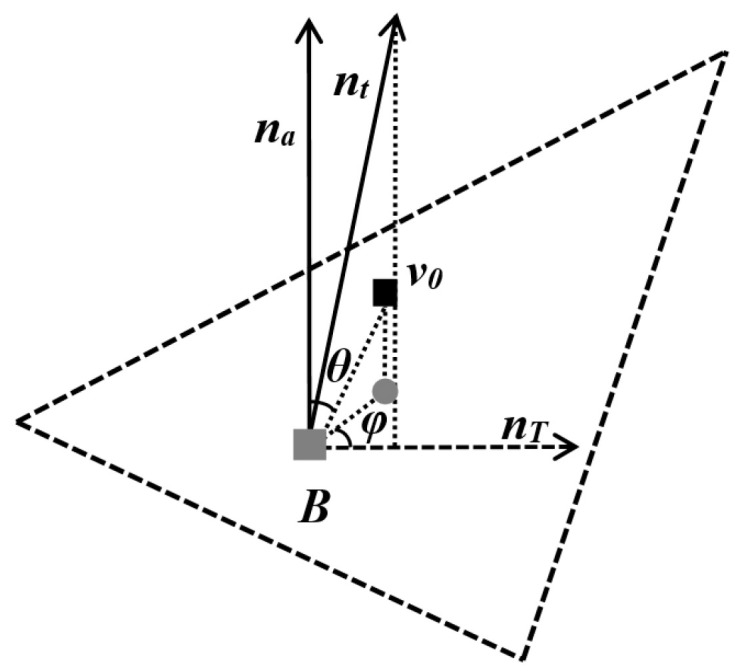
Local spherical coordinates of vertex *v*_0_: ■ vertex of the model; ■ centroid point of the first-ring neighboring points; ● projection point of *v*_0_ onto the projection plane.

Based on the aforementioned normal vectors $n_{a}$ and $n_{t}$, a customized local spherical coordinate system can be established as shown in Figure 3. Taking the centroid *B* (assuming its original coordinates are $\left(x_{0},y_{0},z_{0}\right)$) as the origin of the defined local spherical coordinate system, the plane determined by the centroid *B* and the normal $n_{a}$ is taken as the projection plane. The normal $n_{a}$ serves as the local spherical coordinate system’s *Z*-axis, while the projection $n_{T}$ of the normal $n_{t}$ onto the projection plane serves as the local spherical coordinate system’s *X*-axis. Furthermore, the mean distance *l* from the centroid *B* to the first-ring neighboring points is computed. The ratio between the distance from the model vertex *v*_0_ to the centroid point and *l* is denoted as *r*. The angle between the model vertex *v*_0_ and the positive direction of the *Z*-axis is referred to as *θ*, while the angle with the positive direction of the *X*-axis is denoted as *φ*. The customized spherical coordinate transformation formula is as follows:

(4) $$\left\{\begin{array}{c} r=\frac{\sqrt{{\left(x-x_{0}\right)}^{2}+{\left(y-y_{0}\right)}^{2}+{\left(z-z_{0}\right)}^{2}}}{l}\\ \theta =\cos^{-1}\left(\frac{z-z_{0}}{\sqrt{{\left(x-x_{0}\right)}^{2}+{\left(y-y_{0}\right)}^{2}+{\left(z-z_{0}\right)}^{2}}}\right)\\ \varphi =\left\{\begin{array}{c} \tan^{-1}\left(\frac{y-y_{0}}{x-x_{0}}\right),\;if\;0<\tan^{-1}\left(\frac{y-y_{0}}{x-x_{0}}\right)\leq \pi \\ 2\pi -\tan^{-1}\left(\frac{y-y_{0}}{x-x_{0}}\right),\;if\;\pi <\tan^{-1}\left(\frac{y-y_{0}}{x-x_{0}}\right)\leq 2\pi \end{array}\right. \end{array}\right.$$

Utilizing the previously discussed definitions and calculations, we can determine the local spherical coordinate systems for individual vertices in a 3D mesh model. These coordinate systems serve as the foundation for embedding the first-layer watermark (the meaningful pattern) in the algorithm described in this paper. Prior to embedding, we apply a preprocessing step involving discrete cosine transform encoding to the first-layer watermark. The actual embedding involves the watermark’s information after undergoing discrete cosine transform. As each discrete cosine domain information encompasses the entirety of the original watermark data, in cases where a watermarked model is damaged or subjected to attacks, even if only a limited amount of embedded information can be extracted, the original watermark can still be reasonably recovered.

**Figure 4:**
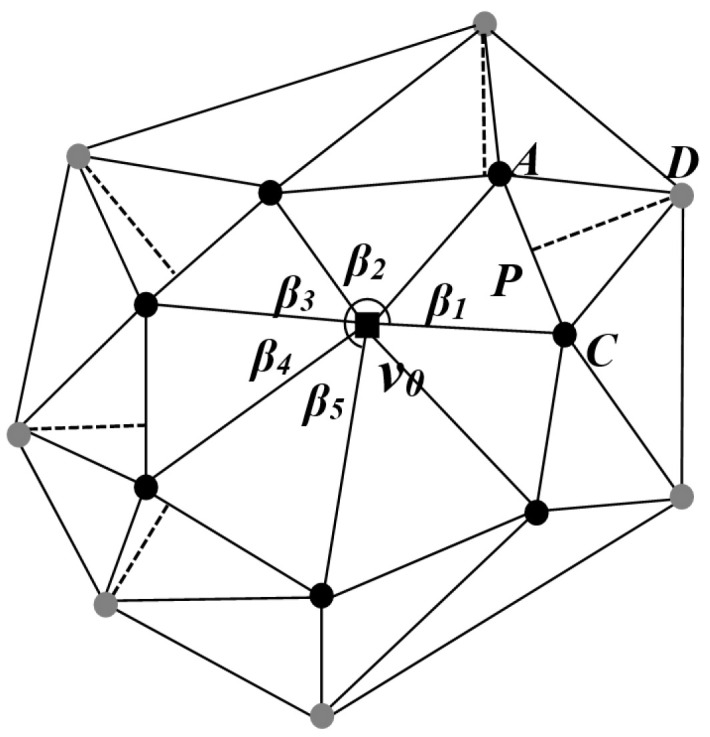
Embedded unit subaggregate of second watermarking: ■ vertex of the model; ● the first-ring neighboring points; ● the second-ring neighboring points.

For the embedding of the second-layer watermark, it is necessary to first define a series of corresponding embedding units. An embedding unit consists of a specific model vertex, its two first-ring neighboring points, and a second-ring neighboring point. Clearly, a single model vertex can correspond to several embedding units. We refer to these embedding units sharing a common model vertex as embedding unit subsets. As shown in Figure 4, a model vertex *v*_0_ along with its first-ring neighboring points *A* and *C*, as well as the corresponding second-ring neighboring point *D*, can form an embedding unit. Here, the line segment *AC* represents the first-ring neighboring edge of vertex *v*_0_, and point *P* denotes the projection of point *D* onto *AC*. The angle between the lines connecting vertex *v*_0_ to its first-ring neighboring points *A* and *C* is denoted as *β*_1_. Similarly, different values of *β* correspond to different index information. Once the index information of the embedding unit is determined, the corresponding watermark binary information is embedded by adjusting the position of the second-ring neighboring point within this unit. When the projection position of the second-ring neighboring point on the first-ring neighboring edge falls within the central region, the corresponding embedded watermark value is 0. If it falls within the side edge regions, the corresponding embedded watermark value is 1. Each embedding unit subset may embed one information value from the first-layer watermark, and possibly multiple information values from the second-layer watermark.

## 3. Watermark Embedding

### 3.1. The Embedding of the First Watermark

**Figure 5:**
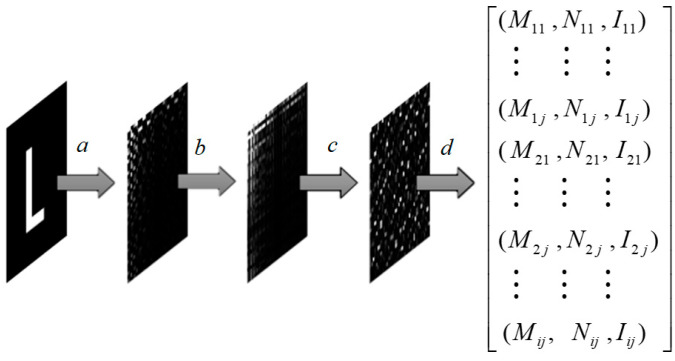
Preprocessing of the first watermark.

First, the watermark image undergoes transform-domain encoding pre-processing, as illustrated in Figure 5. This process involves the following four steps:

(a) A discrete cosine transform is performed on the *m × n* binary watermark image to obtain the corresponding spectral matrix;
(b) The positive and negative coefficient matrices of the transformation matrix are taken as the restoration key, denoted as key 1;
(c) Arnold scrambling is applied to its absolute value matrix, resulting in matrix I, where the scrambling parameters serve as the restoration key, referred to as key 2;
(d) It is necessary to perform index encoding and normalization on the scrambled matrix. As spherical coordinate values are chosen as the embedding carrier, the row and column indices of matrix elements need to be encoded into angle values. The encoding and normalization formulae for the matrix are as follows:
  (5) $$M_{ij}=\frac{m_{ij}}{max\left(m\right)}\cdot \lambda_{1}\pi +\lambda_{2}\pi ;\;N_{ij}=\frac{n_{ij}}{max\left(n\right)}\cdot \lambda_{3}\pi ;\;I_{ij}=\frac{I_{ij}}{max\left(I\right)}$$

Taking into account the watermark’s imperceptibility, the value of *λ*_1_ is set to 0.5, the value of *λ*_2_ is chosen from the range of 0.25 to 0.4, and the value of *λ*_3_ is set to 1. Ultimately, each matrix element is assigned its corresponding attribute values (*M*, *N*, *I*), and these three attribute values are effectively embedded. Additionally, the maximum value of matrix *I* (referred to as *max*(*I*)) is preserved, serving as a recovery value during watermark extraction.

Next, it is necessary to filter out a series of key vertices. The specific filtering criteria are as follows:

1. The centroid *B* of the first-ring neighboring points is calculated for each vertex, and the normal vectors $n_{t}$ and $n_{a}$ of the centroid point are computed. Let the angle between the two normal vectors be denoted as *Φ* = 〈$n_{t},n_{a}$〉. This *Φ* value is used as the first criterion for identifying key vertices, and it should be greater than a certain threshold value $\delta_{1}$. The resulting set of vertices after filtering is denoted as *V*_1_.
2. Since we utilize the ratio of the distance from the vertices to the centroids of their first-ring neighbors, with the mean distance *l* as the embedding carrier, and considering watermark invisibility, it is necessary to impose constraints on the first-ring neighboring points of the vertex set *V*_1_. Let the set of distances between the vertex’s first-ring neighborhood centroid and its first-ring neighbors be denoted as *L*. The ratio of *max*(*L*) to *min*(*L*) should be less than the threshold value $\delta_{2}$. After applying this filtering, the resulting vertex set is denoted as *V*_2_.
3. Feature vertices cannot be adjacent to each other. If they are, all feature vertices in the set *V*_2_ with neighbor relationships are removed. The final filtered set of feature vertices is denoted as *V*_3_.

Then, a custom local spherical coordinate system is established for the feature vertices, as described in Figure 2. Each feature vertex in its corresponding spherical coordinate system possesses three attributes $\left(\theta ,\varphi ,r\right)$. Finally, watermark embedding is performed. During watermark embedding, the watermark attributes (*M*, *N*, *I*) are embedded in descending order of these *I* values. Similarly, the feature vertices are embedded in descending order of the angle *Φ* between the normal vectors of the first-ring neighboring centroid. During the embedding process, the positions of the feature vertices are adjusted to ensure that their attribute values $\left(\theta ,\varphi ,r\right)$ in the corresponding local spherical coordinate systems match the corresponding watermark attribute values (*M*, *N*, *I*). The watermark is traversed and embedded until the entire process is complete.

### 3.2. The Embedding of the Second Watermark

The second watermark involves embedding a binary sequence of length *w*. It should not interfere with the first watermark and must maintain a high level of imperceptibility. During the embedding process, adjustments are made to the positions of the vertices’ second-ring neighbors relative to the first-ring neighboring edges.

First, the second-ring neighboring points (screening points) that can be embedded in the watermark are screened. The vertex set *V*_1_, obtained previously, is used as the base point for embedding the second watermark, and each base point corresponds to several second-ring neighboring points. Considering that the second watermark cannot conflict with the first watermark, and it is convenient to embed the watermark, it is necessary to filter the neighboring points of the second ring. There are four preliminary screening conditions:

(a) It must be connected topologically only to the two first-ring neighboring points of the base point.
(b) The angle *Φ* between the centroid’s normal of its first-ring neighboring points must be less than a certain threshold. This threshold is the same as the one given in step b of the first watermark embedding process, denoted as “$\delta_{1}$”. In other words, it is necessary to exclude the potential embedding points for the first watermark.
(c) The angle *Φ* between the centroid’s normal of the first-ring neighboring points’ own first-ring neighboring points also needs to be less than the threshold “$\delta_{1}$”, given in step b of the first watermark embedding process. This ensures that these points are not first-ring neighbors of vertices that have been embedded with the first watermark.
(d) The triangle created by the base point and its two neighboring points in the first ring (with the edge formed by those two adjacent points as the base) must have both of its base angles measuring less than 90 degrees.

The second-ring neighboring points selected based on the aforementioned conditions are referred to as preliminary filtered points. An embedding unit can be formed by combining the base point, preliminary filtered points, and the two first-ring neighboring points connected to them. This unit comprises four vertices, creating two triangular facets. The base edge of these triangles is formed by connecting the two first-ring neighboring points, and the angle at the base point’s triangle is denoted as *β*. As depicted in Figure 4, the angle *β* falls within the range of $\left(0,\pi \right)$. Considering the relatively limited occurrence of extreme values in the distribution of angle *β*, further refinement is conducted in the selection of filtering points. The points selected for filtering should correspond to *β* values within the range of $\left(a\cdot \pi ,b\cdot \pi \right)$, where “*a*” is slightly greater than 0 and significantly less than 1, while “*b*” is slightly less than 1 and notably greater than 0.

Next, the filtered points are grouped. If “*W*” represents the watermark binary sequence and “*w*” stands for the length of the watermark binary sequence, the filtered points can be divided into “*w*” groups. The range of angle values corresponding to the *i*-th bit of the watermark binary sequence (‘*W_i_*’) is as follows:

(6) $$\left(a\cdot \pi +\frac{b\cdot \pi -a\cdot \pi }{w}i,a\cdot \pi +\frac{b\cdot \pi -a\cdot \pi }{w}\left(i+1\right)\right)\;\left(0\leq i\leq w-1\right)$$

When the angle value corresponding to the filtered point falls within this range, the point is placed in the *i*-th group. Within the same group, the embedding units of the corresponding filtered points are used to embed the corresponding index’s *α* value. The *α* value can be either 0 or 1.

After determining the embedding values *α* for each embedding unit, the second watermark is embedded by adjusting the filtered points within the embedding unit, namely, the second-ring neighbors. As shown in Figure 4, there are two first-ring neighboring points $\left(A,C\right)$ and a second-ring neighboring point *D*. Point *D* is projected onto the first-ring neighbor edge to determine the projection point *P*. If the *α* value and the position of point *P* satisfy Equations (7b) and (7d), no adjustment is needed for the second-ring neighbor point *D*. If the *α* value and the position of point *P* satisfy Equations (7a) and (7c), adjustments are necessary for point *D* to ensure that its position on the first-ring neighboring edge’s projection point *P* meets the requirements.

$$\left\{\begin{array}{c} \frac{min\left(\left|AP\right|,\left|PC\right|\right)}{\left|AC\right|}=0.15\;if\;\alpha =0\;and\frac{min\left(\left|AP\right|,\left|PC\right|\right)}{\left|AC\right|}>0.2\;\left(7a\right)\\ do\;nothing\;if\;\alpha =0\;and\;0\leq \frac{min\left(\left|AP\right|,\left|PC\right|\right)}{\left|AC\right|}\leq 0.2\;\left(7b\right)\\ \frac{min\left(\left|AP\right|,\left|PC\right|\right)}{\left|AC\right|}=0.5\;if\;\alpha =1\;and\frac{min\left(\left|AP\right|,\left|PC\right|\right)}{\left|AC\right|}<0.4\;\left(7c\right)\\ do\;nothing\;if\;\alpha =1\;and\;\frac{min\left(\left|AP\right|,\left|PC\right|\right)}{\left|AC\right|}\geq 0.4\;\left(7d\right) \end{array}\right.$$

During the specific adjustment process, the position coordinates of the projection point *P* are determined based on the *α* value. Then, using point *P* and the first-ring neighboring edge *AC*, a projection plane perpendicular to edge *AC* is established. The second-ring neighboring point *D* is projected onto this plane, yielding a projected point coordinate. The coordinates of point *D* are then replaced with the coordinates of the projected point, completing the watermark embedding for a unit. This process is repeated for all embedding units until the watermark is fully embedded.

## 4. Watermark Detection

To enhance its resilience against various attacks, this paper proposes a dual blind watermark-embedding algorithm. The key idea is that if either of these watermarks is detected, it can be considered as evidence that the model has received effective watermark protection.

### 4.1. Extraction and Detection of the First-Level Watermark

The angle *Φ* between the two normal vectors of each model vertex’s neighboring points within its first ring is calculated. Then, for model vertices where *Φ* exceeds the threshold value $\delta_{1}$ (predefined threshold), a local spherical coordinate system is established. The spherical coordinate values $\left(\theta ,\varphi ,r\right)$ of the model vertex are then calculated within this spherical coordinate system. The subsequent decoding process is as follows:

(8) $$H_{i1}=\frac{\theta_{i}\cdot max\left(M\right)-\lambda_{2}\cdot \pi }{\lambda_{1}\cdot \pi };\;H_{i2}=\frac{\varphi_{i}\cdot max\left(n\right)}{\lambda_{3}\cdot \pi };\;H_{i3}=\frac{r_{i}}{\rho }\cdot \max\left(I\right)$$

An *m × n* zero matrix *O*, where each matrix element has three attribute values $\left(M,N,0\right)$ (*M* = 1,2,3…*m*, *N* = 1,2,3…*n*), is defined. When the difference between the row and column indices of one zero matrix element and the decoded values of the vertex spherical coordinates is less than the threshold values *σ*1 and *σ*2, respectively, the zero value can be replaced. Here, *σ*1 and *σ*2 are taken as 0.01 and 0.005, respectively:

(9) $$O_{mn}=H_{i3}\;if\;\left|M-H_{i1}\right|<\sigma 1\;and\;\left|N-H_{i2}\right|<\sigma 2$$

Next, the matrix *O* is restored using the key 2 for Arnold permutation, followed by applying the positive and negative coefficients using the key 1. Then, an inverse discrete cosine transform is performed to obtain the extracted watermark image *O*. Finally, the correlation between the extracted watermark image matrix *O* and the original watermark image (represented as *S*) is calculated using the following correlation equation:

(10) $$cor1=\frac{\sum_{i=1}^{m}\sum_{j=1}^{n}S\left(i,j\right)O\left(i,j\right)}{\sqrt{\sum_{i=1}^{m}\sum_{j=1}^{n}S{\left(i,j\right)}^{2}}\sqrt{\sum_{i=1}^{m}\sum_{j=1}^{n}O{\left(i,j\right)}^{2}}}$$

When the value of *cor*1 exceeds the specified threshold, it can be concluded that the model has embedded the first watermark. Conversely, if the value is below this threshold, it is considered that the first watermark has not been embedded.

### 4.2. Extraction and Detection of the Second-Level Watermark

First, vertices are selected that satisfy the conditions as base points. The selection criteria for base points are the same as those in step b of the first watermark-embedding process, which means that the angle between the two normal vectors of a vertex’s centroid and its first-ring neighbors, denoted as *Φ*, should be greater than a given threshold value $\delta_{1}$. Once the base points are selected, the potential second watermark-embedding points among their second-ring neighbors can be identified. Each base point contains several second-ring neighbors, but not all of them contain information about the second watermark. The specific selection method for these neighbors is the same as the filtering process during the embedding step.

After the filtering process, the filtered points are grouped. The size of the angle *β* in the triangle where the base point resides is used as the basis for grouping, effectively serving as an index for the watermark binary sequence. Once the groups and the embedding units within each group are determined, the evaluation of watermark values can be conducted. As shown in Figure 4, let us assume that $\left(v_{0},A,C,D\right)$ represents a filtered unit. The next step involves projecting the second-ring neighboring point *D* onto the first-ring neighboring edge, determining the projection point *P*. The relative position of the projection point *P* is then used to ascertain whether the watermark is embedded and to determine the embedding value *α*, as illustrated below:

(11) $$\left\{\begin{array}{c} \alpha =0\;if\;\frac{min\left(\left|AP\right|,\left|PC\right|\right)}{\left|AC\right|}\leq 0.2\\ \alpha =1\;if\;\frac{min\left(\left|AP\right|,\left|PC\right|\right)}{\left|AC\right|}\geq 0.4 \end{array}\right.$$

After performing binary extraction on all units within each group, a “voting decision” process is carried out to determine the binary value for each group (1, 2, …, *i*, …, *w*), thereby obtaining the binary sequence *W*1, as depicted below:

(12) $$\left\{\begin{array}{c} W1_{i}=1\;if\;number\left(0\right)<number\left(1\right)\\ \;W1_{i}=0\;if\;number\left(0\right)>number\left(1\right) \end{array}\right.$$

The original watermark binary sequence is denoted as *W*, with $\bar{W}$ being its mean. The extracted watermark binary sequence is denoted as *W*1, with $\bar{W1}$ being its mean. The formula for calculating the correlation coefficient is as follows:

(13) $$cor2=\frac{\sum_{i=1}^{w}\left(W1\left(i\right)-\bar{W1}\right)\left(W\left(i\right)-\bar{W}\right)}{\sqrt{\sum_{i=1}^{w}{\left(W1\left(i\right)-\bar{W1}\right)}^{2}}\sqrt{\sum_{i=1}^{w}{\left(W\left(i\right)-\bar{W}\right)}^{2}}}$$

When the value of *cor*2 is greater than the specified threshold, it can be concluded that the model has embedded the second watermark.

## 5. Experimental Results and Analysis

**Figure 6:**
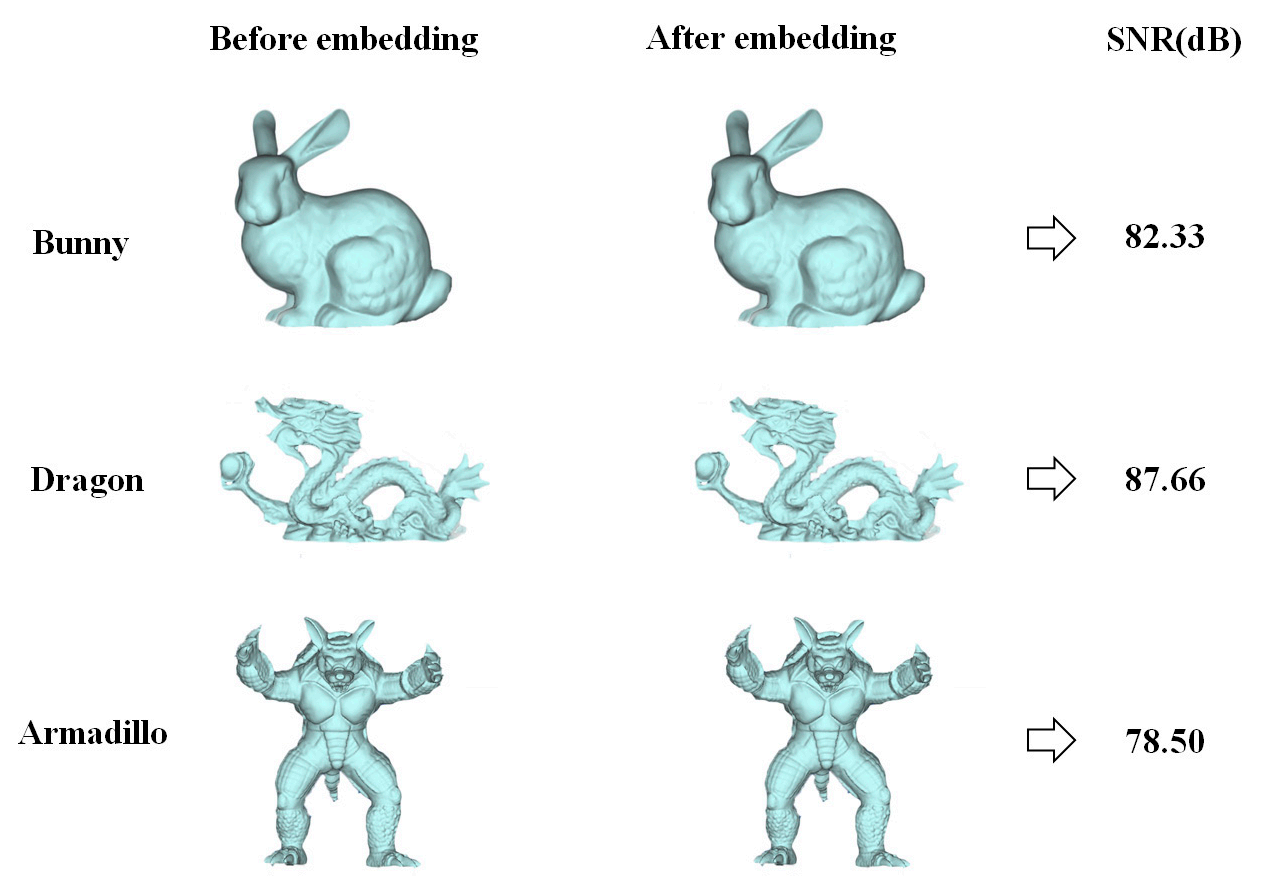
Experimental 3D models.

In this paper, the first watermark selected for the primary layer has a size of 40 × 40 pixels and consists of a binary image depicting the letter “L.” The secondary watermark, on the other hand, comprises a random binary sequence of 32 bits. The experimental models which we employed are simplified versions of the bunny, dragon, and armadillo 3D mesh models from Stanford University’s 3D Mesh Repository. As shown in Figure 6, the three models on the left represent the models before watermark embedding, while the one in the middle represents the model after watermark embedding. It is challenging to visually observe any significant changes in the model. The calculated signal-to-noise ratios (SNR) caused by embedding the watermarks were 82.33, 87.66, and 78.50 dB, respectively. This indicates that after watermark embedding, the imperceptibility of the watermark was good.

After embedding the watermarks into the models, it was necessary to establish appropriate thresholds to determine the validity of the extracted watermarks. Based on experience, this study set the relevance threshold at 0.3. The following section presents the results and analysis of the watermark robustness attack experiments which were conducted.

(1) Affine transformation and vertex reordering attacks

Affine transformation attacks involve operations such as translation, rotation, and scaling applied to a model, which alter the coordinate positions of the model’s vertices. The first watermark in this study, being embedded within the local spherical coordinate system of the model’s vertices, naturally possessed immunity against translation, rotation, and uniform scaling. Consequently, the first watermark could be extracted in its entirety. The index value of the second watermark was embedded within angular information. As translation, rotation, and uniform scaling attacks do not affect angular information, the second watermark could also be extracted completely. Furthermore, the embedding of the two watermarks was not correlated with the sequence of model vertices. Therefore, even when subjected to vertex reordering attacks, the dual watermark information could still be fully extracted.

(2) Cropping attack

The first watermark was embedded into the information in the transform domain by adjusting the feature vertices in the spatial domain. Even with a small amount of extracted information, the overall contour of the watermark image could be approximately reconstructed. As a result, the first watermark exhibited strong resistance to cropping attacks. Similarly, the second watermark, due to the distribution of its watermark information across multiple subunits for each bit, also demonstrated robust resistance to cropping attacks. While both watermarks exhibited strong resilience against cropping, the first watermark served as a visually interpretable watermark, making it more meaningful compared to the second watermark (Table 1).

(3) Simplification Attack

The essence of a simplification attack lies in minimizing the number of vertices and triangles while preserving the model’s features. In this type of attack, certain points and triangles may be removed. The first watermark was relatively sensitive to vertex loss and perturbation, making it weaker in terms of countering simplification attacks. On the other hand, the second watermark was comparatively less sensitive than the first one and possessed stronger resistance against simplification attacks (Table 2).

(4) Noise Attack

Uniform random noise was added to the coordinates of watermark-embedded model vertices, where the magnitude of noise was defined as the ratio of the length of the noise vector to the distance from the mesh vertex to the mesh center. The first watermark was relatively sensitive to vertex perturbations, making it less resistant to noise attacks. In contrast, the second watermark’s index values corresponded to angles, and the watermark values corresponded to projected positions. Both had more leniency, and the final watermark value was determined by statistics. Therefore, the second watermark exhibited stronger resistance to noise attacks. Comparing the data in references [5,13,28], this algorithm demonstrated a higher capability to withstand noise attacks (Table 3).

(5) Smoothing Attack

Smoothing attacks lead to the loss of surface details in 3D models, and the greater the degree of smoothing, the more severe the loss of details becomes. In this algorithm, the resistance of the first watermark against smoothing attacks was weaker, while the second watermark exhibited stronger robustness. The experimental results indicate that the algorithm’s ability to resist smoothing attacks is also influenced by the inherent roughness of the model itself. Models like the dragon and armadillo are rougher compared to the bunny model, making smoothing attacks more impactful on these models. Consequently, the detected relevance of the second watermark was lower for the dragon and armadillo models than for the bunny model (Table 4).

(6) Combined Attacks

In existing dual blind watermarking algorithms, although the design of dual watermarks has, to some extent, enhanced resistance against individual attacks, the ability to resist combined attacks is significantly lacking. For instance, as demonstrated in references [13,28], their algorithms were unable to extract either watermark in the case of combined cropping and noise attacks. In contrast, the algorithm proposed in this paper was still able to effectively extract the second watermark when subjected to certain levels of such attacks (Table 5).

(7) Comparison with Similar Algorithms

This algorithm is a blind watermarking technique, meaning that watermark extraction does not require the involvement of the original model. Similar to this algorithm, there have been works such as references [8,14,26,27,28]. The approach in reference [8] is a single blind watermarking method, capable of resisting affine transformation and cropping attacks, yet able to withstand limited attack types. Both references [14,28] proposed dual blind watermarking schemes, expanding the range of attack resistances, but they failed to counter simplification, smoothing, and noise combined with cropping attacks. Reference [26] employed the skewness measure of the spherical angle as the resilient feature; however, it was unable to resist the cropping attacks. As the watermark data were drawn by modifying the vertex control of the structure in [27], it could not prevent noise, smoothing, or mesh simplification. In contrast, the algorithm presented in this paper, aside from being vulnerable to non-uniform scaling attacks, exhibited a certain resistance against various attacks, including affine transformation, cropping, noise, simplification, smoothing, and combined attacks. A comparison between this algorithm and the other three is illustrated in Table 6.

## 6. Conclusions

This paper introduces a dual blind watermarking algorithm that demonstrates robustness for 3D mesh models. The algorithm embeds two distinct watermarks using two different embedding methods within the 3D model. These watermarks remain mutually independent and do not interfere with each other. The resulting watermarked model maintains excellent invisibility, and watermark extraction does not require the original model, thus achieving blind detection. The experimental results show that the algorithm displays a certain level of resistance against various attacks, including affine transformation, cropping, noise, simplification, smoothing, and combined attacks. In comparison to similar previous blind watermarking algorithms, this algorithm extends the range of attacks it can withstand. However, it should be noted that neither watermark 1 or watermark 2 can be effectively extracted under non-uniform scaling attacks, which represents a limitation of this algorithm and a subject for future research.

## Figures and Tables

**Table 1:** Cropping attack.

Cropping Rate	Bunny		Dragon		Armadillo	
	*cor*1	*cor*2	*cor*1	*cor*2	*cor*1	*cor*2
15%	0.8404	1	0.8127	1	0.8216	0.9113
30%	0.7663	1	0.7223	1	0.7867	0.9010
45%	0.7012	1	0.6501	1	0.6908	0.9000
60%	0.5417	1	0.5564	1	0.6071	0.8547
75%	0.4867	1	0.4491	1	0.4675	0.8081
90%	0.3739	0.9344	0.2175	1	0.3714	0.8014

**Table 2:** Simplification attack.

Simplification Ratio	Bunny		Dragon		Armadillo	
	*cor*1	*cor*2	*cor*1	*cor*2	*cor*1	*cor*2
5%	0.0717	0.9344	0.0888	1	0.0443	1
10%	0.0949	0.8207	0.0160	0.9344	0.0247	0.9315
15%	0.0627	0.5238	0.0132	0.7014	0.0079	0.6625
20%	0.0062	0.3477	0.0035	0.6101	0.0017	0.5554
25%	0.0013	0.2328	0.0290	0.4667	0.0012	0.3615

**Table 3:** Noise attack.

Noise Intensity	Bunny		Dragon		Armadillo	
	*cor*1	*cor*2	*cor*1	*cor*2	*cor*1	*cor*2
0.5%	0.0252	1	0.0688	1	0.0670	1
1%	0.0040	1	0.0075	1	0.0413	0.9344
1.5%	0.0022	1	0.0461	1	0.0052	0.9344
2%	0.0057	1	0.0296	1	0.0208	0.7896

**Table 4:** Smooth attack.

Level of Smoothing	Bunny		Dragon		Armadillo	
	*cor*1	*cor*2	*cor*1	*cor*2	*cor*1	*cor*2
1	0.0008	0.9344	0.0271	0.6102	0.0073	0.6958
2	0.0025	0.7237	0.0039	0.3710	0.0037	0.5057
3	0.0257	0.6798	0.0460	0.3031	0.0138	0.3391
4	0.0255	0.7237	0.0107	0.2659	0.0060	0.2051

**Table 5:** Combined attack.

Combined Attacks	Bunny		Dragon		Armadillo	
	*cor*1	*cor*2	*cor*1	*cor*2	*cor*1	*cor*2
2% noise + 15%cropping	0.0023	1	0.0078	1	0.0212	0.9344
2% noise + 30% cropping	0.0015	1	0.0081	1	0.0209	0.9344
2% noise + 45% cropping	0.0021	1	0.0071	0.9344	0.0200	0.7984
2% noise + 60% cropping	0.0008	0.9344	0.0065	0.8150	0.0012	0.7229

**Table 6:** Comparison of algorithms.

Types of Attacks	Can It Resist Attacks?					
	Method Proposed	Reference [8]	Reference [14]	Reference [28]	Reference [26]	Reference [27]
Affine Transformation Attack	Yes	Yes	Yes	Yes	Yes	Yes
Non-uniform Scaling Attack	No	Yes	No	Yes	Yes	Yes
Cropping Attack	Yes	Yes	Yes	Yes	No	Yes
Noise Attack	Yes	Yes	No	Yes	Yes	No
Simplification Attack	Yes	No	No	No	Yes	No
Smoothing Attack	Yes	No	No	No	Yes	No
Noise and Cropping Combined Attack	Yes	No	No	No	No	No

## Data Availability

The data underlying the results presented in this paper are not publicly available at this time, but may be obtained from the authors upon reasonable request.
